# The *w*Mel strain of *Wolbachia* Reduces Transmission of Zika virus by *Aedes aegypti*

**DOI:** 10.1038/srep28792

**Published:** 2016-07-01

**Authors:** Matthew T. Aliota, Stephen A. Peinado, Ivan Dario Velez, Jorge E. Osorio

**Affiliations:** 1 Department of Pathobiological Sciences, University of Wisconsin-Madison, Madison, WI, USA; 2 Programa de Estudio y Control de Enfermedades Tropicales (PECET), Universidad de Antioquia, Medellin, A1226, Colombia

## Abstract

Zika virus (ZIKV) is causing an explosive outbreak of febrile disease in the Americas. There are no effective antiviral therapies or licensed vaccines for this virus, and mosquito control strategies have not been adequate to contain the virus. A promising candidate for arbovirus control and prevention relies on the introduction of the intracellular bacterium *Wolbachia* into *Aedes aegypti* mosquitoes. This primarily has been proposed as a tool to control dengue virus (DENV) transmission; however, evidence suggests *Wolbachia* infections confer protection for *Ae. aegypti* against other arboviruses. At present, it is unknown whether or not ZIKV can infect, disseminate, and be transmitted by *Wolbachia-*infected *Ae. aegypti*. Using *Ae. aegypti* infected with the *w*Mel strain of *Wolbachia* that are being released in Medellin, Colombia, we report that these mosquitoes have reduced vector competence for ZIKV. These results support the use of *Wolbachia* biocontrol as a multivalent strategy against *Ae. aegypti*-transmitted viruses.

Zika virus (ZIKV) is an arbovirus that belongs to the family *Flaviviridae*. It currently is causing an explosive outbreak of febrile disease in the Americas. In humans, ZIKV infection typically causes a mild and self-limiting illness known as Zika fever, which often is accompanied by maculopapular rash, headache, and myalgia^1^,^2^. During the current outbreak, a causal relationship has been established between prenatal ZIKV infection and microcephaly and other serious brain anomalies^3^,^4^,^5^,^6^. Prior to this, ZIKV existed in relative obscurity with only sporadic confirmed human infections until the end of the last century^7^. The virus is believed to have originated in Africa, where it still circulates enzootically among unknown vertebrate hosts (presumably nonhuman primates), and is transmitted by arboreal *Aedes* mosquitoes^8^,^9^,^10^. These cycles lead to occasional outbreaks of spillover infection in Africa, but most human cases around the globe result from ZIKV emergence into a human-mosquito cycle involving *Aedes aegypti*^11^ and/or other urban or peri-urban *Aedes* species, e.g., *Aedes albopictus* and *Aedes hensilli*^12^,^13^,^14^.

Despite the continued spread of the virus, there remain no effective antiviral therapies or licensed vaccines. Thus, with its continued invasion of the new world, the only tools presently available to combat Zika target mosquito populations, mostly with insecticides and larval source reduction. To date, these strategies have not prevented invasion of this virus into new locales and have not been adequate to control the virus upon arrival. A promising candidate for arbovirus control and prevention relies on the introduction of the intracellular bacterium *Wolbachia* into *Ae. aegypti* mosquitoes. *Wolbachia* is a maternally-inherited intracellular bacterium that is present in numerous insect species worldwide, including mosquitoes, butterflies, beetles, ants, and bees^15^,^16^. Interestingly, *Ae. aegypti* has no native *Wolbachia* symbionts. The *w*MelPop strain of the bacterium therefore was introduced into *Ae. aegypti* with the intent to control dengue virus (DENV) transmission by shortening the lifespan of female mosquitoes^17^. This alone could have had a potentially major impact on disease transmission by greatly reducing the number of females old enough to transmit the virus^18^; however, that strain of *Wolbachia* no longer is being considered for biocontrol because infected mosquitoes displayed reduced fitness in small-scale field releases^19^. Serendipitously, it also was discovered that some *Wolbachia* interfere with viruses and other microbes in the same host. For example, certain *Wolbachia* variants (e.g., the *w*Mel strain) partially block DENV transmission without greatly impacting *Ae. aegypti* fitness^20^,^21^,^22^. The *w*Mel strain conferred protection against chikungunya virus (CHIKV) and yellow fever virus (YFV) in *Ae. aegypti* as well^23^,^24^,^25^. These findings lend optimism that *Wolbachia* biocontrol represents a significant new development in the fight against arbovirus transmission. Indeed, because DENV, CHIKV, YFV, and now ZIKV^26^,^27^,^28^ co-circulate in many parts of the tropics *Wolbachia* biocontrol could potentially be used as a multivalent strategy against all four of these *Ae. aegypti*-transmitted viruses. But the ability of ZIKV to infect, disseminate, and be transmitted by *Wolbachia-*infected *Ae. aegypti* has not been evaluated.

Accordingly, we assessed vector competence for ZIKV in *w*Mel-infected and *w*Mel-free *Ae. aegypti* from Medellin, Colombia. This was done because medium-scale deployments of *w*Mel-infected *Ae. aegypti* began in the DENV metropolitan area of Medellin in the spring of last year (2015) [see www.eliminatedengue.com/colombia], and now ZIKV co-circulates with DENV in Colombia. The data presented herein provide information on the effectiveness of Colombian *Wolbachia*-infected *Ae. aegypti* in blocking the transmission of ZIKV, as well as describe a biologically relevant model for studying ZIKV transmission dynamics (i.e., exposure to virus was accomplished by feeding on a viremic host) that does not rely on animal blood spiked with cultured virus. These data argue for the expansion of this technology to ZIKV in South and Central America and are useful in the broader context of ZIKV-mosquito interactions in the Americas.

## Results

**Figure 1:**
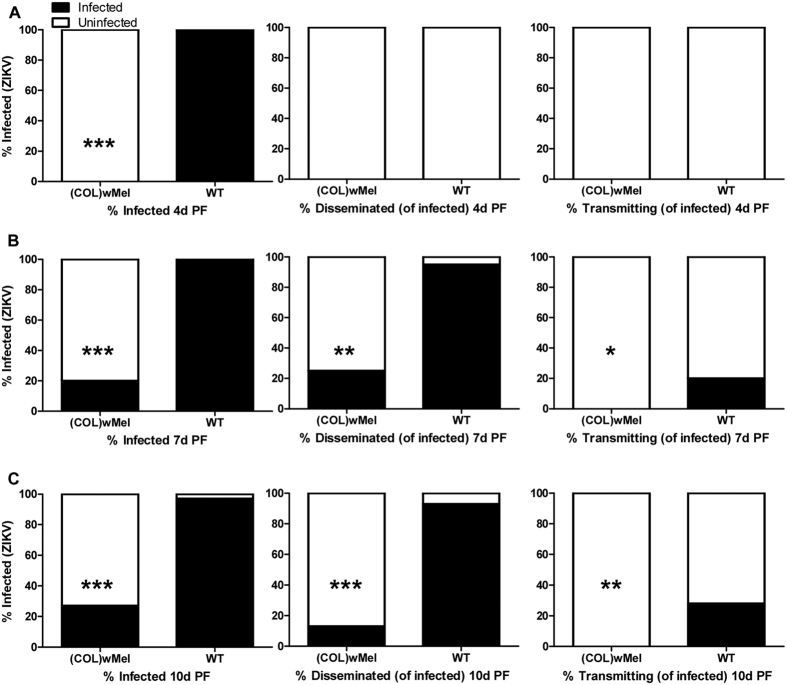
Vector competence of WT and (COL)*w*Mel mosquitoes orally infected with 6.02 log_10_ PFU/ml of ZIKV. Mosquitoes were allowed to feed on ZIKV-infected mice and were examined at days 4, 7, and 10 post feeding to determine infection, dissemination, and transmission efficiencies. Infection efficiency corresponds to the proportion of mosquitoes with virus-infected bodies among the tested ones. Dissemination efficiency corresponds to the proportion of mosquitoes with virus-infected legs, and transmission efficiency corresponds to the proportion of mosquitoes with infectious saliva among those infected. *significant reduction in infection rates (*p < 0.05, **p < 0.01, ***p < 0.001) (**A**). Four days post feeding (n = 20) (**B**). Seven days post feeding (n = 20) (**C**). Ten days post feeding (n = 30).

**Figure 2:**
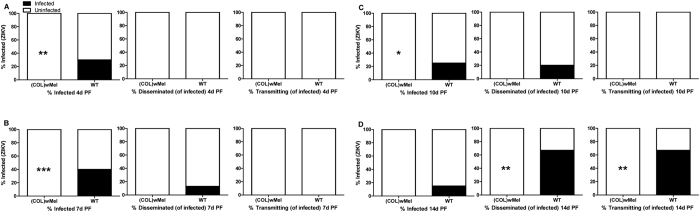
Vector competence of WT and (COL)*w*Mel mosquitoes orally infected with 4.74 log_10_ PFU/ml of ZIKV. Mosquitoes were allowed to feed on ZIKV-infected mice and were examined at days 4, 7, 10, and 14 post feeding to determine infection, dissemination, and transmission efficiencies. Infection efficiency corresponds to the proportion of mosquitoes with virus-infected bodies among the tested ones. Dissemination efficiency corresponds to the proportion of mosquitoes with virus-infected legs, and transmission efficiency corresponds to the proportion of mosquitoes with infectious saliva among those infected. *significant reduction in infection rates (*p < 0.05, **p < 0.01, ***p < 0.001) (**A**). Four days post feeding (n = 20) (**B**). Seven days post feeding (n = 20) (**C**). Ten days post feeding (n = 20) (**D**). Fourteen days post feeding (n = 20).

**Figure 3:**
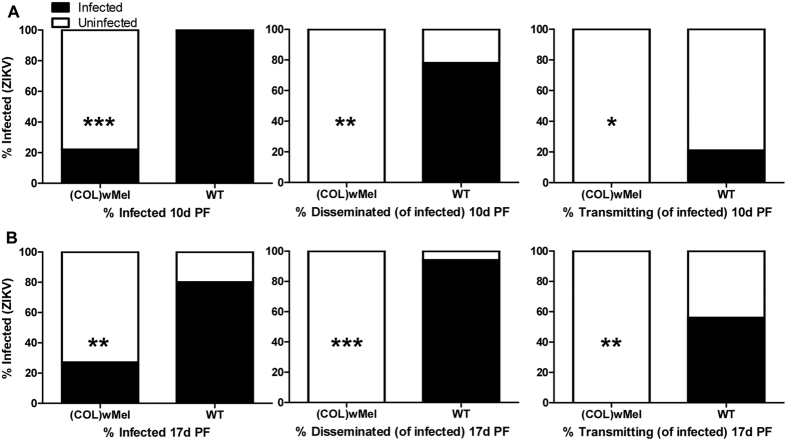
Vector competence of WT and (COL)*w*Mel mosquitoes orally infected with 8.00 log_10_ PFU/ml of ZIKV. Mosquitoes were exposed to a ZIKV-infected bloodmeal via water-jacketed membrane feeder and were examined at days 10 and 17 post feeding to determine infection, dissemination, and transmission efficiencies. Infection efficiency corresponds to the proportion of mosquitoes with virus-infected bodies among the tested ones. Dissemination efficiency corresponds to the proportion of mosquitoes with virus-infected legs, and transmission efficiency corresponds to the proportion of mosquitoes with infectious saliva among those infected. *significant reduction in infection rates (*p < 0.05, **p < 0.01, ***p < 0.001) (**A**). Ten days post feeding (n = 18 for (COL)*w*Mel and n = 19 for WT) (**B**). Seventeen days post feeding (n = 15 for (COL)*w*Mel and n = 20 for WT).

Here, we verified that the phenotype of reduced vector competence existed in *Wolbachia*-infected laboratory colonies of Colombian *Ae. aegypti* for ZIKV. Adult, female, mosquitoes were exposed to infectious bloodmeals containing ZIKV and mosquitoes that ingested blood containing virus were assayed for infection, dissemination, and transmission potential at 4, 7, 10, 14, and 17 days (d) post feeding (PF). ZIKV infection status was confirmed by plaque assay to differentiate infectious from non-infectious virus. As expected, infection, dissemination, and transmission rates were high for WT exposed to blood containing ZIKV. In contrast, (COL)*w*Mel that ingested blood containing ZIKV displayed poor peroral vector competence as compared to WT. In fact, there was a significant reduction (Exact Unconditional Test) in ZIKV infection status as compared to WT at all timepoints assayed and across replicates, and (COL)*w*Mel remained non-infective over the duration of experimentation (Figs 1, 2, 3). Similar findings also were recently reported by Dutra *et al*.^29^.

These data also are informative for a more accurate appraisal of ZIKV transmission by *Ae. aegypti.* To better understand ZIKV transmission dynamics, we assessed the subtleties of infection in WT over time following infectious bloodmeals that were in agreement with viremias detected in patients in Colombia where the mean ± standard deviation serum viral load was 4.42 log_10_ viral copies/ml ± 1.02 (n = 10, range = 2.73–5.84 log_10_ viral copies/ml). After feeding on ZIKV-infected mice with moderate viremia (6.02 log_10_ PFU/ml), 100% of WT mosquitoes had established infections at 4 d PF, but none of the mosquitoes disseminated virus nor was infectious virus detected in the saliva (Fig. 1A). At 7 d PF, most mosquitoes disseminated virus (95%) and a moderate number also were infective (Fig. 1B). By 10 d PF, the transmission rate had increased to 28% (Fig. 1C). In contrast, WT that fed on ZIKV-infected mice with low viremia (4.74 log_10_ PFU/ml) did not have detectable virus in the saliva until 14 d PF (Fig. 2A–C).

To investigate whether or not higher viral titers in the bloodmeal increase the probability of mosquito infection, mosquitoes were exposed to a relatively high viremic bloodmeal (8.00 log_10_ PFU/ml) via water-jacketed membrane feeder maintained at 36.5 °C^30^. Surprisingly, infection, dissemination, and transmission rates were almost identical for both (COL)*w*Mel and WT exposed to viremic mice as compared to mosquitoes exposed to a membrane feeder spiked to a higher viremia (Figs 1 and 3). At 10 d PF, the infection rate for mouse-exposed (COL)*w*Mel was 27% (Fig. 1C) and for membrane feeder-exposed (COL)*w*Mel it was 22% (Fig. 3A). Likewise, the transmission rate for mouse-exposed WT was 28% and for membrane feeder-exposed WT it was 21%. By 17 d PF, the transmission rate peaked at 56% for membrane feeder-exposed WT (Fig. 3B). In comparison, we observed a transmission rate of 67% at 14 d PF in WT that were exposed to mice with relatively low viremias (Fig. 2C). These data highlight the importance of investigating vector competence by allowing mosquitoes to feed on a viremic host because membrane feeding influenced the magnitude of the observed effect.

## Discussion

There is a paucity of data available on vector competence for ZIKV. The few studies available have primarily focused on two urban vectors, *Ae. aegypti* and *Ae. albopictus*^13^,^31^, but other *Aedes* species may be important vectors depending on the specific geographic location^12^,^32^,^33^. The first ZIKV vector competence study with *Ae. aegypti* was conducted in 1956^34^ and it was demonstrated that *Ae. aegypti* was capable of transmitting ZIKV for ten weeks. Interestingly, ZIKV was not detectable on days 5 and 10 PF. Another study involving transmission to a rhesus monkey demonstrated that the extrinsic incubation period (EIP) of ZIKV was about 15 days^35^. Both of these results differ from what we report here. We were able to detect ZIKV infection in *Ae. aegypti* as early as 4 d PF and the EIP of ZIKV ranged from 7–14 days depending on viremia of the host. This may indicate differences between the propensity of African versus Asian lineage ZIKV to infect and replicate in *Ae. aegypti* but will require further validation in the laboratory.

More recently, it was demonstrated that Singapore’s *Ae. aegypti* were highly capable of transmitting ZIKV^36^. In contrast, *Ae. aegypti* strains from Kedougou and Dakar (Senegal) were not competent to transmit ZIKV^32^. Similar results recently were observed with *Ae. aegypti* strains from the Americas, which were susceptible to ZIKV infection but had unexpectedly low transmission potential^31^. Here we demonstrated that a strain of *Ae. aegypti* from Medellin, Colombia was highly susceptible to ZIKV infection and were competent in their potential to transmit the virus to a new host. It should be noted that *Ae. aegypti* with poor competence but high population density, have been capable of sustaining arbovirus outbreaks^37^. And, that the geographic variation in oral susceptibility of mosquitoes of the same species to different viruses is not unusual^38^,^39^,^40^. This argues for continued studies (both experimental and epidemiological) assessing interactions between differing *Ae. aegypti*-ZIKV combinations.

The history of ZIKV has been reminiscent of CHIKV, which re-emerged out of Africa and now circulates on all inhabited continents and represents a major global health problem. Not surprisingly, ZIKV has emerged in Colombia^2^, likely following the path of CHIKV, which reached the country in August 2014^41^ and now both viruses co-circulate with DENV and YFV. Current vector control measures have been insufficient in preventing invasion of ZIKV and CHIKV into the country or controlling it after invasion. Although primarily deployed as a biocontrol tool for DENV, evidence suggests that *Wolbachia* can limit infection in *Ae. aegypti* with other arboviruses^24^,^25^; therefore, *Wolbachia-*infected *Ae. aegypti* could potentially be used to simultaneously control DENV, CHIKV, YFV, and ZIKV. As a result, we evaluated whether Colombian mosquitoes infected with the *w*Mel strain of *Wolbachia* reduced ZIKV transmission potential. Studies like this one, aimed at assessing the potential effectiveness of *Wolbachia* biocontrol against ZIKV, will help inform the viability of using this technology as a multivalent strategy for multiple *Ae. aegypti-*transmitted arboviruses. This novel approach is already being tested in five countries around the globe (Australia, Brazil, Colombia, Indonesia, and Vietnam) against related arboviruses, so these results warrant further exploration, both in the laboratory and the field, on the feasibility of expanding this technology beyond DENV and informing whether *Wolbachia* biocontrol can be used to supplement or replace existing vector control strategies.

## Methods

### Ethics Statement

This study was carried out in strict accordance with recommendations set forth in the National Institutes of Health *Guide for the Care and Use of Laboratory Animals*. All animals and animal facilities were under the control of the School of Veterinary Medicine with oversight from the University of Wisconsin Research Animal Resource Center. The protocol was approved by the University of Wisconsin Animal Care and Use Committee (Approval #V01327).

### Cells and viruses

African Green Monkey kidney cells (Vero; ATCC #CCL-81) were grown in Dulbecco’s modified Eagle medium (DMEM) supplemented with 10% fetal bovine serum (FBS; Hyclone, Logan, UT), 2 mM L-glutamine, 1.5 g/l sodium bicarbonate, 100 U/ml of penicillin, 100 μg/ml of streptomycin, and incubated at 37 °C in 5% CO_2_. *Aedes albopictus* mosquito cells, (C6/36; ATCC #CRL-1660) were maintained in MEM supplemented with 10% FBS, 2 mM L-glutamine, 1.5 g/l sodium bicarbonate, 0.1 mM non-essential amino acids, 100 U/ml of penicillin, 100 μg/ml of streptomycin, and incubated at 28 °C in 5% CO_2_. ZIKV strain PRVABC59 (GenBank:KU501215), originally isolated from a traveler to Puerto Rico with three rounds of amplification on Vero cells, was obtained from Brandy Russell (CDC, Ft. Collins, CO). Virus stocks were prepared by inoculation onto a confluent monolayer of C6/36 mosquito cells.

### Mosquito strains and colony maintenance

*Ae. aegypti* used in this study were maintained at the University of Wisconsin-Madison as previously described^42^. Two lines of mosquitoes were used in this study. Wild type (WT) mosquitoes (not infected with *Wolbachia*) were established from several hundred eggs collected from 41 ovitraps placed around the municipality of Bello (commune 1), a northwest suburb of Medellin, Colombia. The *Wolbachia*-infected ((COL)*w*Mel; infected with the *w*Mel strain of *Wolbachia pipientis*) mosquito line was generated by crossing wild-caught *Aedes aegypti* with a *w*Mel-infected laboratory strain of *Ae. aegypti* using a scheme of mating to field-collected males as developed by Yeap *et al.*^43^ to generate a Colombian genetic background *Wolbachia*-infected mosquito. The *w*Mel-infected laboratory population of *Ae. aegypti* originated from eggs provided by Scott O’Neill (Monash University, Victoria Australia). Briefly, females from the *w*Mel-infected laboratory population were backcrossed to wild-caught males until generation five at which time the lines were closed (restricting matings to mosquitoes from within the line) with the population size maintained at several thousand adults. An outcrossed line then was established by continuous backcrossing laboratory mosquitoes to the progeny of wild-caught *Ae. aegypti* from the Bello region (same location as eventual release zones). The WT line was derived from material collected from the same 41 ovitraps distributed throughout the suburb of Bello, Colombia. This line has never had any previous contact with *Wolbachia*-infected mosquitoes. Wild-caught males from the Bello region were routinely collected and introduced into the (COL)*w*Mel and WT colony cages after each generation to prevent inbreeding effects and to ensure the genetic homogeneity of these two lines. *Wolbachia* infection status was routinely verified using PCR with primers specific to the *IS5* repeat element^20^.

### Exposure to infective bloodmeal

Mosquitoes were exposed to ZIKV by feeding on isoflurane anesthetized ZIKV-infected *Ifnar−/−* mice. These mice have abrogated type I interferon signaling and as a result develop lethal infection and a high viremia. *Ifnar−/−* mice on the C57BL/6 background were obtained from Eva Harris (University California-Berkeley, Berkeley, CA) and were bred in the pathogen-free animal facilities of the University of Wisconsin-Madison School of Veterinary Medicine. Groups of three-week-old mixed sex mice were used for mosquito exposures. Mice were infected in the left, hind foot pad with 10^6^ plaque forming units (PFU) of ZIKV in 50 μl of animal diluent (AD: 1% heat-inactivated FBS in Dulbecco’s PBS). Uninfected mosquitoes (both WT and (COL)*w*Mel) were allowed to feed on mice two or three days post infection at which time sub-mandibular blood draws were performed and serum was collected to verify viremia. Mice fed upon two days post infection (biological replicate one) yielded an infectious bloodmeal concentration of 6.02 log_10_ PFU/ml ± 0.67 (mean ± standard deviation; n = 4) and mice fed upon three days post infection (biological replicate two) yielded an infectious bloodmeal concentration of 4.74 log_10_ PFU/ml ± 0.06. A third biological replicate was performed, whereby mosquitoes were exposed to a ZIKV-infected bloodmeal via water-jacketed membrane feeder maintained at 36.5 °C^30^. Bloodmeals consisted of defibrinated sheep blood (HemoStat Laboratories) and fresh virus supernatant, yielding an infectious bloodmeal concentration of 8.0 log_10_ PFU/ml (bloodmeal titer was determined after feeding).

### Vector Competence

Infection, dissemination, and transmission rates were determined for individual mosquitoes and sample sizes were chosen using long established procedures^25^,^44^,^45^. Briefly, three- to six-day-old female mosquitoes were sucrose starved for 14 to 16 hours prior to exposure to mice or membrane feeder. Mosquitoes that fed to repletion were randomized, separated into cartons in groups of 20–30, and maintained on 0.3 M sucrose in an environmental chamber at 26.5 °C ± 1 °C, 75% ± 5% relative humidity, and with a 12 hour photoperiod within the Department of Pathobiological Sciences BSL3 Insectary facility at the University of Wisconsin-Madison. All samples were screened by plaque assay on Vero cells. Dissemination was indicated by virus-positive legs. Transmission was defined as release of infectious virus with salivary secretions, i.e., the potential to infect another host, and was indicated by virus positive-salivary secretions.

### Viral Quantification

All ZIKV screens from mosquito tissues and titrations for virus quantification from mouse serum or virus stocks were completed by plaque assay on Vero cell cultures. Duplicate wells were infected with 0.1 ml aliquots from serial 10-fold dilutions in growth media and virus was adsorbed for one hour. Following incubation, the inoculum was removed, and monolayers were overlaid with 3 ml containing a 1:1 mixture of 1.2% oxoid agar and 2X DMEM (Gibco, Carlsbad, CA) with 10% (vol/vol) FBS and 2% (vol/vol) penicillin/streptomycin. Cells were incubated at 37 °C in 5% CO_2_ for four days for plaque development. Cell monolayers then were stained with 3 ml of overlay containing a 1:1 mixture of 1.2% oxoid agar and 2X DMEM with 2% (vol/vol) FBS, 2% (vol/vol) penicillin/streptomycin, and 0.33% neutral red (Gibco). Cells were incubated overnight at 37 °C and plaques were counted. Viral RNA from human serum samples from Colombia was quantified by qRT-PCR using the primers and probe designed by Lanciotti *et al.*^46^. The RT-PCR was performed using the iTaq^TM^ Universal One-Step RT-qPCR kit (BioRad, Hercules, CA) on an iCycler® instrument (BioRad, Hercules, CA). Primers and probe were used at final concentrations of 600 nm and 100 nm respectively. Cycling conditions were as follows: 37 °C for 15 min, 50 °C for 30 min and 95 °C for 2 min, followed by 50 cycles of 95 °C for 15 sec and 60 °C for 1 min. Virus concentration was determined by interpolation onto an internal standard curve made up of a 7-point dilution series of *in vitro* transcribed RNA.

### Statistical Analysis

Infection, dissemination, and transmission rates were analyzed using an Exact unconditional test^47^. This test replaces Fisher’s exact test. It also is exact but has the advantage of being more sensitive in detecting differences (i.e., its statistical power is higher) in the case of sample sizes less than 100^47^. Each biological replicate consisted of mosquitoes from distinct generations to take into account stochastic variations.

## Additional Information

**How to cite this article**: Aliota, M. T. *et al.* The *w*Mel strain of *Wolbachia* Reduces Transmission of Zika virus by *Aedes aegypti*. *Sci. Rep.* **6**, 28792; doi: 10.1038/srep28792 (2016).
